# Mutation of GDP-Mannose-4,6-Dehydratase in Colorectal Cancer Metastasis

**DOI:** 10.1371/journal.pone.0070298

**Published:** 2013-07-29

**Authors:** Kotarosumitomo Nakayama, Kenta Moriwaki, Taku Imai, Shinichiro Shinzaki, Yoshihiro Kamada, Kohei Murata, Eiji Miyoshi

**Affiliations:** 1 Department of Molecular Biochemistry and Clinical Investigation, Osaka University Graduate School of Medicine, Osaka, Japan; 2 Department of Surgery, Suita Municipal Hospital, Osaka, Japan; University of Barcelona, Spain

## Abstract

Fucosylation is a crucial oligosaccharide modification in cancer. The known function of fucosylation in cancer is to mediate metastasis through selectin ligand-dependent processes. Previously, we found complete loss of fucosylation in the colon cancer cell line HCT116 due to a mutation in the GDP-fucose synthetic enzyme, GDP-mannose-4,6-dehydratase (GMDS). Loss of fucosylation led to escape of cancer cells from tumor immune surveillance followed by tumor progression and metastasis, suggesting a novel function of fucosylation in tumor progression pathway. In the present study, we investigated the frequency of GMDS mutation in a number of clinical colorectal cancer tissue samples: 81 samples of primary colorectal cancer tissue and 39 samples of metastatic lesion including liver and lymph node. Four types of deletion mutation in GMDS were identified in original cancer tissues as well as metastatic lesions. The frequency of GMDS mutation was slightly higher in metastatic lesions (12.8%, 5/39 samples) than in original cancer tissues (8.6%, 7/81 samples). No mutation of the GMDS gene was observed in normal colon tissues surrounding cancer tissues, suggesting that the mutation is somatic rather than in the germline. Immunohistochemical analysis revealed complete loss of fucosylation in three cases of cancer tissue. All three cases had GMDS mutation. In one of three cases, loss of fucosylation was observed in only metastatic lesion, but not its original colon cancer tissue. These data demonstrate involvement of GMDS mutation in the progression of colorectal cancer.

## Introduction

Fucosylation is one of the most important oligosaccharide modifications in cancer and inflammation [1]. Fucosylation is regulated by various fucosyltransferases, guanosine 5′-diphosphate (GDP)-fucose synthetic enzymes, and GDP-fucose transporters. Most GDP-fucose is synthesized by the de novo pathway in which GDP-mannose is transformed into GDP-fucose by GDP-mannose-4,6-dehydratase (GMDS) and GDP-4-keto-6-deoxymannose-3,5-epimerase-4-reductase (FX) [2]–[4]. Several antibodies that recognize fucosylated glycoproteins or glycolipids in sera of patients with cancer have long been used as tumor markers [5]. The alpha-fetoprotein (AFP)-L3 fraction, which is fucosylated AFP, has also been used clinically as a tumor marker for hepatocellular carcinoma since 1996 in Japan and since 2005 in the United States [6]. In general, fucosylation levels are increased during carcinogenesis of several kinds of cancer [7], [8]. Previously, however, we found that complete loss of fucosylation due to deletion mutation of GMDS gene allowed colon cancer cells to escape from natural killer cell-mediated tumor surveillance through modulation of tumor necrosis factor-related apoptosis-inducing ligand (TRAIL) signaling [9], suggesting that a novel metastatic pathway dependent on loss of fucosylation. GMDS mutation has been observed in colon (HCT116, LS174T, NCI-H716) and gastric (SCH) cancer cell lines as well as in human colon and ovarian cancer tissues [9]. Interestingly, GMDS mutation was not found in any adjacent normal tissues, suggesting that GMDS mutation was somatic. If loss of fucosylation is critical for tumor metastasis during colorectal cancer progression, the frequency of GMDS mutation would likely be increased in metastatic lesions. In this study, we investigated the frequency of GMDS mutation in metastatic colorectal cancer tissues such as liver and lymph node.

## Materials and Methods

### Ethics Statement

The protocol and informed consent were approved by institutional review boards at Osaka University Graduate School of Medicine. Written informed consent was obtained from all patients, and the study was conducted in accordance with the Helsinki Declaration.

### Tissue Samples

Thirty-one samples of metastatic liver cancer, 2 samples of metastatic other cancers (gastric cancer, thyroid cancer) and 81 samples of the original colon cancer tissues derived from patients with colorectal cancer who underwent primary resection at the Department of Surgery at Osaka Medical Center for Cancer and Cardiovascular Diseases from 1995 to 2005 were stored at −80°C until used. Six samples from metastatic lymph nodes from patients with colorectal cancer who underwent primary resection at the Department of Surgery at Suita Municipal Hospital from 2010 to 2012 were also used in this study. Clinical parameters of patients in this study are summarized in Table 1. Some of the cancer tissues were embedded in paraffin and used for immunohistochemical analysis. These studies were approved by the institutional ethics committee of the Osaka University Hospital.

**Table 1:** Clinical parameters of patients in this study.

	Cases (n = 117)
**Sex (men/women)**	69/48
**Age (MEAN ± SD)**	66.2±11.5
**Clinical stage**	
0	4 (3%)
I	16 (14%)
II	41 (35%)
IIIa	12 (10%)
IIIb	6 (5%)
IV	38 (32%)
**Primary tumor site**	
Colon	106 (91%)
Rectum	11 (9%)

### Screening of GMDS Mutation with Reverse Transcription-polymerase Chain Reaction (RT-PCR) Analysis

Total RNA was extracted from frozen tissues according to a standard protocol using TRIzol (Invitrogen, Carlsbad, CA). The extracted RNA was reverse-transcribed using Super Script™ III reverse transcriptase and the Oligo dT primer (Invitrogen). Using synthesized cDNA, PCR was performed with KOD-Plus-DNA polymerase (TOYOBO, Osaka, Japan). The PCR primers for GMDS were as follows: F, 5′-GCAAGCTTAAAATGGCACACGCACCGGCAC-3′ and R, 5′-GCGGATCCTCAGGCATTGGGGTTTGTC-3′. Glyceraldehydes-3-phosphate dehydrogenase (GAPDH) was used as an internal control, and the following PCR primers were used to amplify GAPDH: F, 5′-AACGGGAAGCTTGTCATCAAT-3′ and R, 5′-GCCAGTGAGCTTCCCGTTCA-3′. Sequence analysis was performed with an ABI PRISM 3100 genetic analyzer (Applied Biosystems, Foster City, CA).

### Immunohistochemical Studies

Cancer and normal colon tissues were fixed with 10% formalin/phosphate-buffered saline (PBS) and stored as paraffin-embedded samples. The 4-µm tissue sections were de-waxed, and endogenous peroxidase activity was blocked by treatment with 0.3% hydrogen peroxide in methanol for 10 min. After washing twice with PBS, the samples were incubated with Tris buffered saline and Tween 20 containing 5% bovine serum albumin overnight at 4°C. The samples were incubated with biotinylated *Aleuria aurantia* lectin (AAL; 2.0 µg/ml) or rabbit anti-GMDS antibody (0.3 µg/ml) for 1 hour at room temperature. Samples were washed three times with PBS and subsequently incubated with the ABC kit (Vector Labs, Burlingame, CA) for AAL staining or with Dako Cytomation Envision+ System- HRP Labeled Polymer Anti-Rabbit antibody (Dako, Glostrup, Denmark) for GMDS staining at room temperature for 30 min. After samples were washed three times with PBS, positive staining was visualized using diaminobenzidine (Dako).

## Results

### GMDS Mutation in Colorectal Cancer

**Figure 1:**
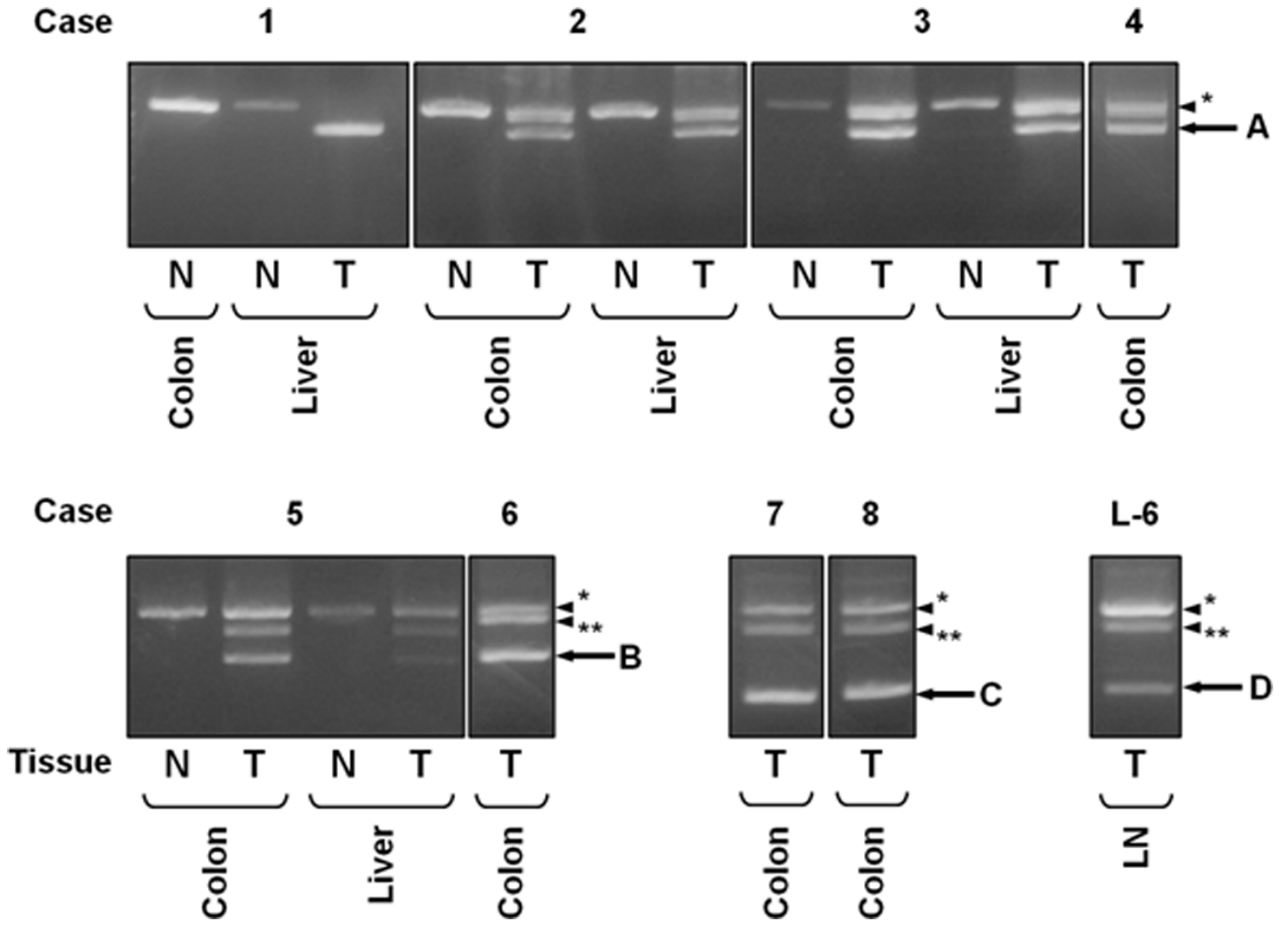
GMDS mutations in the original colorectal cancers and metastatic lesions. GMDS mutations were observed in seven cases of original colorectal cancer tissues and five cases of metastatic lesions. Arrows indicate bands representing GMDS mutation: deletion of exons 2–4 (A, 876 bp), 5–7 (B, 693 bp), 2–7 (C, 450 bp), and 3–7 (D, 495 bp). Arrowheads indicate wild type GMDS (*) and non-specific (**) bands. L-6 indicates one of the cases with metastatic lymph nodes. N, adjacent normal tissue; T, tumor; LN, lymph node.

To examine the frequency of GMDS mutation in original and metastatic colorectal cancers, total RNA was extracted from 81 samples of human original colorectal cancer tissues, 39 samples of metastatic cancer tissues, and adjacent normal colon tissues and was subjected to RT-PCR analysis. Four shorter PCR products were found in several original and metastatic cancer tissues (Fig. 1). Detailed sequence analysis revealed different types of deletion of GMDS exons: exons 2–4, 5–7, 2–7, and 3–7. Two of these mutations, deletion of exons 5–7 and exons 2–4, were identical to those in the HCT116 and SCH cell lines, respectively. Deletions of exons 2–7 and 3–7 of the GMDS gene represent novel mutations identified in this study. The GMDS mutations in the metastatic lesions were consistent with those from the original colorectal cancer tissues. Interestingly, the homozygote of GMDS mutation without normal type of GMDS transcript was found in one metastatic liver cancer tissue (case 1 in Fig. 1). The frequency of GMDS mutation in metastatic lesions was 12.8% (5/39 samples): 12.9% (4/31 samples) in liver, 16.7% (1/6 samples) in lymph node, and 0% (0/2 samples) in other organs (Table 2). A slightly lower frequency 8.6% (7/81 samples) of GMDS mutation was observed in the original cancer tissues compared to their metastatic lesions even though the difference was not statistically significant (*p*<0.10, by χ^2^ test). No GMDS mutation was observed in 24 samples of adjacent normal colon tissues.

**Table 2:** Frequency of GMDS mutation in original and metastatic lesions of human colorectal cancer.

Tissue		Samples	Frequency (%)
**Adjacent normal colon**		0/24	0.0
**Primary cancer**		7/81	8.6
**Metastasis**		5/39	12.8
**Breakdown of metastasis**	**Lymph node**	1/6	16.7
	**Liver**	4/31	12.9
	**Others**	0/2	0.0

### Immunohistochemistry

**Figure 2:**
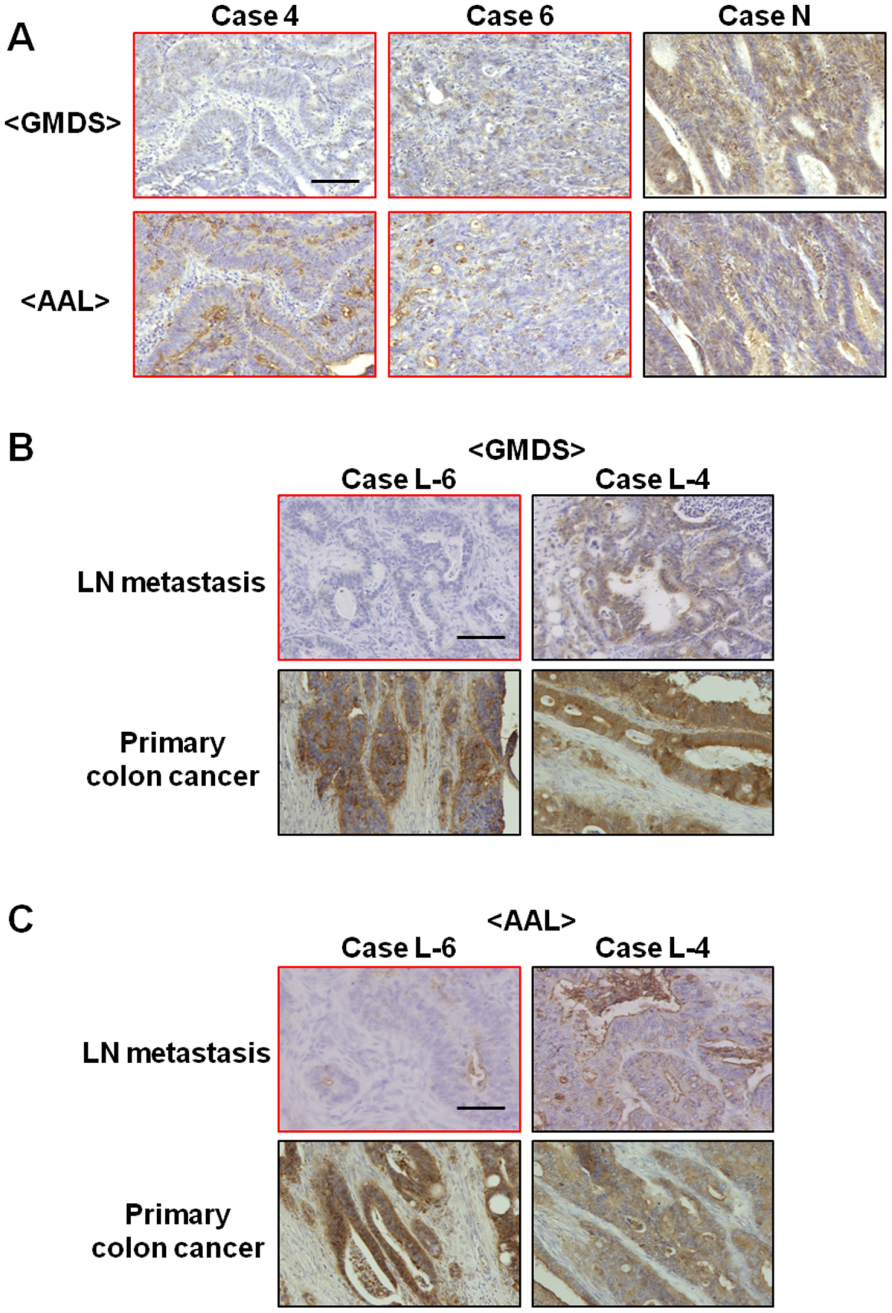
Immunohistochemical analyses of original colorectal cancers and their metastatic lymph nodes. Thirty-three cases of original colorectal cancer tissues and four cases of their metastatic lymph nodes were stained with anti-GMDS antibody and AAL. (A) Cases 4 and 6, which depict the original colorectal cancer with GMDS mutation, but not case N, which did not have GMDS mutation, showed negative staining for both GMDS and AAL. (B, C) In case L-6, which did harbor GMDS mutation, the metastatic lymph node was not stained for GMDS (B) and AAL (C) despite positive staining for both GMDS and AAL in the original colorectal cancer tissue sample. Case L-4 without GMDS mutation showed positive staining for GMDS and AAL in both the original and metastatic legions. Bar indicates 100 µm. LN, lymph node.

To examine cellular fucosylation level in these cancer tissues, 33 cases of original colorectal cancer tissues and four cases of their metastatic lymph nodes were stained with anti-GMDS antibody and a fucosylated glycan-binding lectin, AAL. RT-PCR analyses showed heterozygous GMDS mutation in five of the 33 cases. Twenty-eight colorectal cancer tissues without GMDS mutation showed positive staining for both GMDS and AAL. Representative pictures are shown in Fig. 2A (case-N). In contrast, two of five cases of the original cancer with GMDS mutation showed negative staining for both GMDS and AAL (case 4 and 6 in Fig. 2A). Interestingly, one of five cases with GMDS mutation showed negative staining for both GMDS and AAL in the metastatic lymph node in spite of positive staining in its original colon cancer tissue (case L-6 in Fig. 2B and C).

## Discussion

In previous our study, the GMDS mutation was identified by gDNA sequencing in two out of 100 cases of human colorectal cancer tissue and by RT-PCR analysis in five out of 10 cases of microdissected human ovarian cancer tissue. In this study, we further demonstrated the GMDS mutation in several human original and metastatic colorectal cancer tissues. The frequency of GMDS mutation was slightly higher in metastatic lesions (12.8%) than in the original cancer tissues (8.6%). Interestingly, in one case (L-6), loss of fucosylation was observed in the metastatic lymph node but not in its original cancer tissue (Fig. 2B and C). These results suggest that GMDS mutation is involved in the progression of colorectal cancer. The number of cases with GMDS mutation was not sufficient to examine the statistical correlation between GMDS mutation and disease activity with any certainty. Further analysis with more number of samples will be required to determine the correlation between GMDS mutation and colon cancer progression. Four out of nine patients with GMDS mutation (case 1–8 and case L-6) were subjected to surgical operation within 3 years after diagnosis. Thus, a follow-up study is also required to investigate recurrence of metastasis.

Although most of the GMDS mutations observed in this study were heterozygous, homozygous deletion mutation was observed in one metastatic liver cancer tissue (case 1, Fig. 1). Since cancer tissues consist of a variety of cells, including not only cancer cells but also interstitial cells, it is difficult to demonstrate whether cancer cells harbor a heterozygous or homozygous type of mutation by RT-PCR analysis using whole cancer tissues. Thus, the possibility that cancer cells have a homozygous GMDS deletion mutation in tissues in which the heterozygous deletion mutation was observed remains. In fact, expression of GMDS and fucosylated glycans were barely detected by immunohistochemical analysis using anti-GMDS antibody and AAL in three of five cases with a heterozygous GMDS mutation, suggesting that cancer cells in these tissues may actually have homozygous GMDS mutation.

Expression levels of the GMDS gene in cancer tissues are affected by not only gene mutation but also by transcriptional regulation. Although some of the glycosylation-related genes were reported to be epigenetically regulated [10], [11], expression of GMDS was not altered by treatment with agents that modulating epigenetic DNA structure via DNA methylation or histone acetylation, suggesting that epigenetic effects do not play a major role in regulating GMDS gene expression [12] (data not shown). Further studies addressing gene expression regulation of the GMDS gene are warranted.

Among many fucosylation-related genes, mutation of the fucosyltransferases FUT1, 2, and 3, which catalyze α1–2 or 1–3/4 fucosylation, has been reported in patients with rare blood types [13], [14]. In addition, GDP-fucose transporter has also been reported to be responsible for leukocyte adhesion deficiency type II which is a rare recessive syndrome characterized by growth and mental retardation and severe immunodeficiency [15]–[17]. No mutation of fucosylation-related genes has been reported in human cancer tissues before. GMDS is the first fucosylation-related gene that was found to be mutated in cancer tissues. Next-generation DNA sequence analysis may be a useful tool to determine why GMDS mutation occurs in several kinds of cancers.

High fucosylation level in cancer cells was demonstrated to increase metastasis through upregulating expression of sialyl Lewis A and X, selectin ligands, on cell surface [10]. In contrast, our results show that loss of fucosylation also increased metastasis even in the absence of selectin ligands. Cancer metastasis progresses through many steps: escape from immune cells, invasion, angiogenesis, intravasation, homing to metastatic tissues, extravasation, and colonization [18]. Fucosylation could have a different role in each step. Fucosylation in cancer cells needs to be tightly regulated and its dysregulaiton will cause further cancer progression and metastasis. In conclusion, the present study demonstrated that GMDS mutation should be involved in the progression of colorectal cancer. Next-generation DNA sequence analysis may give us more information about GMDS mutation in colorectal cancer.
